# MicroRNA-378 Suppressed Osteogenesis of MSCs and Impaired Bone Formation via Inactivating Wnt/β-Catenin Signaling

**DOI:** 10.1016/j.omtn.2020.07.018

**Published:** 2020-07-15

**Authors:** Lu Feng, Jin-fang Zhang, Liu Shi, Zheng-meng Yang, Tian-yi Wu, Hai-xing Wang, Wei-ping Lin, Ying-fei Lu, Jessica Hiu Tung Lo, Da-hai Zhu, Gang Li

**Affiliations:** 1 Department of Orthopaedics & Traumatology, Li Ka Shing Institute of Health Sciences and Lui Che Woo Institute of Innovative Medicine, Faculty of Medicine, The Chinese University of Hong Kong, Prince of Wales Hospital, Shatin, Hong Kong SAR, P.R. China; 2 Key Laboratory of Orthopaedics and Traumatology, The First Affiliated Hospital of Guangzhou University of Chinese Medicine, The First Clinical Medical College, Guangzhou University of Chinese Medicine, Guangzhou, P.R. China; 3 Laboratory of Orthopaedics & Traumatology, Lingnan Medical Research Center, Guangzhou University of Chinese Medicine, Guangzhou, P.R. China; 4 Trauma Center, Zhongda Hospital, School of Medicine, Southeast University, No. 87 Ding Jia Qiao, Nanjing, P.R. China; 5 School of Medicine, Southeast University, No. 87 Ding Jia Qiao, Nanjing, P.R. China; 6 Department of Orthopaedic Surgery, Shanghai Jiao Tong University Affiliated Sixth People’s Hospital, Shanghai, P.R. China; 7 Central Laboratory, The Affiliated Jiangning Hospital with Nanjing Medical University, Nanjing, Jiangsu 211100, P.R. China; 8 The State Key Laboratory of Medical Molecular Biology, Institute of Basic Medical Sciences, Chinese Academy of Medical Sciences and School of Basic Medicine, Peking Union Medical College, Beijing, P.R. China; 9 The CUHK-ACC Space Medicine Centre on Health Maintenance of Musculoskeletal System, The Chinese University of Hong Kong Shenzhen Research Institute, Shenzhen, P.R. China

**Keywords:** miR-378, mesenchymal stem cells, osteogenesis, Wnt/β-catenin signaling, bone formation

## Abstract

MicroRNAs (miRNAs) have been reported to serve as silencers to repress gene expression at post-transcriptional levels. Multiple miRNAs have been demonstrated to play important roles in osteogenesis. MicroRNA (miR)-378, a conserved miRNA, was reported to mediate bone metabolism and influence bone development, but the detailed function and underlying mechanism remain obscure. In this study, the miR-378 transgenic (TG) mouse was developed to study the role of miR-378 in osteogenic differentiation as well as bone formation. The abnormal bone tissues and impaired bone quality were displayed in the miR-378 TG mice, and a delayed healing effect was observed during bone fracture of the miR-378 TG mice. The osteogenic differentiation of mesenchymal stem cells (MSCs) derived from this TG mouse was also inhibited. We also found that miR-378 mimics suppressed, whereas anti-miR-378 promoted osteogenesis of human MSCs. Two Wnt family members, Wnt6 and Wnt10a, were identified as bona fide targets of miR-378, and their expression was decreased by this miRNA, which eventually induced the inactivation of Wnt/β-catenin signaling. Finally, the short hairpin (sh)-miR-378-modified MSCs were locally injected into the fracture sites in an established mouse fracture model. The results indicated that miR-378 inhibitor therapy could promote bone formation and stimulate the healing process *in vivo*. In conclusion, miR-378 suppressed osteogenesis and bone formation via inactivating Wnt/β-catenin signaling, suggesting that miR-378 may be a potential therapeutic target for bone diseases.

## Graphical Abstract

**Figure:**
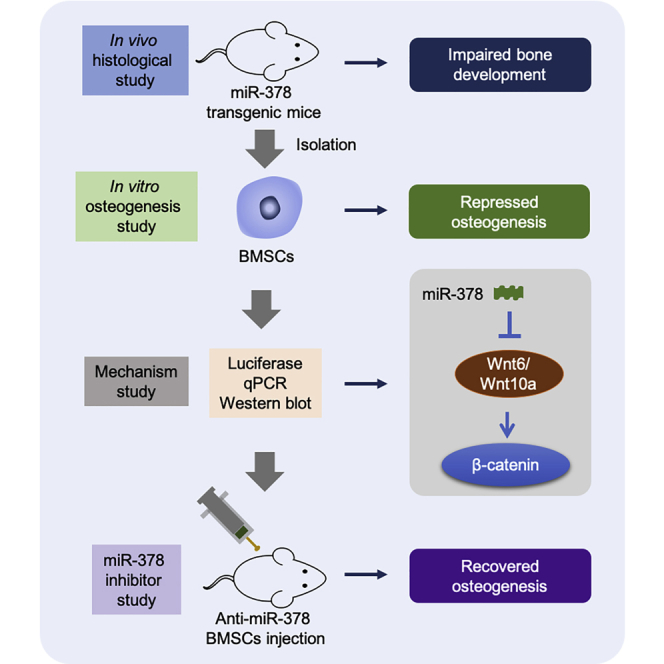

## Teaser

Feng and his colleagues identified impaired bone development and BMSCs osteogenesis in miR-378 transgenic mice. They characterized that miR-378 isoforms directly repress two Wnt family members’ expression, which leads to deactivation of Wnt/β-catenin signaling during osteogenesis. They also demonstrated the effect of miR-378 inhibitor therapy on promoting bone formation in vivo.

## Introduction

Bone regeneration is very important for the recovery of some diseases, including osteoporosis and bone-fracture trauma. It is a multiple-step and multiple-gene involved complex process, including osteoblast-mediated bone formation and osteoclast-mediated bone resorption. During this process, mesenchymal stem cells (MSCs) gradually differentiate into osteoblasts, which produce a variety of extracellular matrix and thus induce the initiation of bone formation. Hence, the improvement of the osteoblast differentiation of MSCs is crucial for the development of a therapeutic strategy for bone diseases.

Over the past few years, microRNAs (miRNAs) have emerged as gene silencers to suppress gene expression at post-transcriptional levels. Multiple miRNAs have been demonstrated to play important roles in biological activities, including osteogenic differentiation and skeletal development. For example, microRNA (miR)-26a, miR-29b, miR-125b, miR-133, miR-135, and miR-196a have been reported to be involved in osteogenesis.^1^, ^2^, ^3^, ^4^, ^5^ Our previous reports also demonstrated that miR-20a and -20b promoted, whereas miR-637 suppressed, osteoblast differentiation.^6^^,^^7^ Therefore, miRNAs have been considered as potential candidates to mediate osteogenic differentiation and may act as therapeutic targets for bone regeneration.

miR-378, which is derived from a hairpin RNA precursor, has been reported to promote bone morphogenetic protein 2 (BMP2)-induced osteoblast differentiation in C2C12 cells^8^ and attenuate high glucose-suppressed osteogenic differentiation in a preosteoblastic cell line,^9^ demonstrating the promoting effect on osteoblast. However, another group reported that miR-378 suppressed osteogenic differentiation of MC3T3-E1 cells.^10^ Therefore, the function of miR-378 in osteogenesis remains intriguing.

In this study, abnormal bone tissues and impaired bone quality were exhibited in the miR-378 transgenic (TG) mice, and a delayed bone formation and fracture healing were also found in these TG mice. Moreover, we found that miR-378 overexpression suppressed, whereas its antagonists promoted, osteogenic differentiation, suggesting that miR-378 may play a significant role in osteoblast differentiation and bone regeneration. Furthermore, two Wnt family members, Wnt6 and Wnt10a, were identified as targets of miR-378, and their expression was decreased by miR-378, which eventually suppressed Wnt/β-catenin signaling. Therefore, miR-378 inhibited osteoblast differentiation and impaired bone formation, suggesting that it may be a potential therapeutic target for bone diseases.

## Results

### Abnormal Bone Tissues and Impaired Bone Quality Were Observed in miR-378 TG Mice

**Figure 1:**
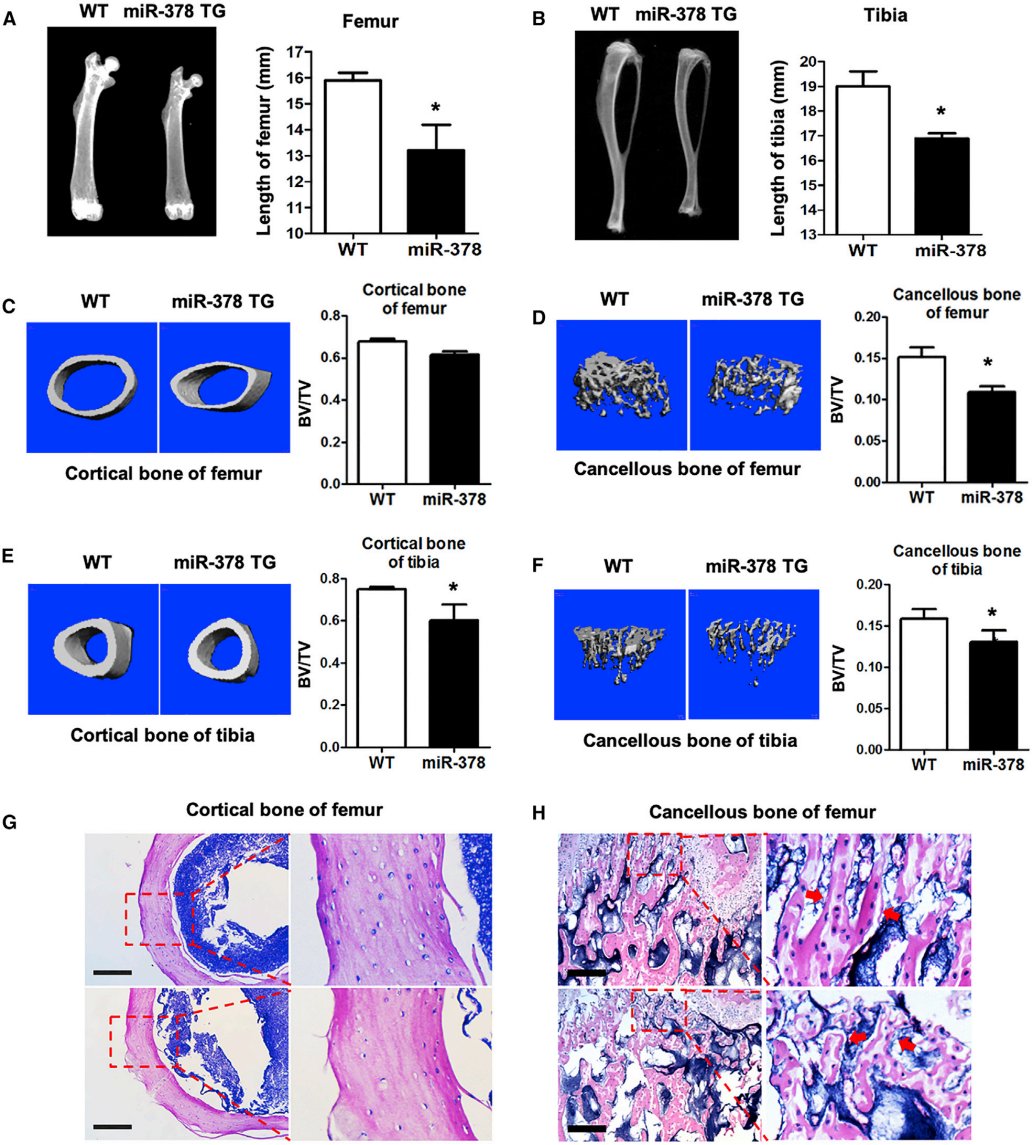
Abnormal Bone Tissues and Impaired Bone Quality Were Observed in miR-378 TG Mice (A and B) Bone phenotype of femurs (A) and tibia (B) of miR-378 TG mice and their wild-type (WT) mice were examined by digital radiography. (C–F) Representative microarchitecture 3D images of cortical (C and E) and trabecular (D and F) bone of femur (C and D), as well as tibia (E and F). BV/TV of the cortical and trabecular bone all showed that the bone mass was significantly decreased in the miR-378 TG mice (age: 3 months; n = 10; ∗p < 0.05 versus WT group). (G) H&E staining of transverse and coronal plane of femurs revealed thicker cortices, smoother edge of cortical bone, and more paralleled alignment of osteocytes in the WT mice than those in the miR-378 TG group. Scale bars, 400 μm. (H) H&E staining of the sagittal plane of the metaphyseal region of femurs revealed more disrupted bone microarchitecture and homeostasis in cancellous bone of the miR-378 TG mice femur. Scale bars, 100 μm.

To investigate the function of miR-378 in bone formation, we compared the bone tissues from miR-378 TG mice and that from their wild-type (WT) mice. By digital radiography examination, the bone size and length of the femur (Figure 1A), tibia (Figure 1B), head (Figure S1A for side view and Figure S1B for vertical view), tail (Figure S1C), and spine (Figure S1D for front view and Figure S1E for side view) were all decreased in the miR-378 TG mice. We further investigated the microarchitecture of femurs and tibia by micro-computed tomography (micro-CT) examination. The representative three-dimensional (3D) images showed a significant loss of bone mass in trabecular bone of femur (Figure 1D), as well as cortical and trabecular bone of tibia (Figures 1E and 1F) in miR-378 TG mice. The femur cortical bone mass is lower in miR-378 TG mice without significance. Furthermore, hematoxylin and eosin (H&E) staining of femurs showed the thinner growth plate, less smooth edge of cortical bone, and less paralleled alignment of osteocytes in TG mice (Figure 1G, red arrow) and disturbed trabecular bone structure around the metaphyseal region of femurs in miR-378 TG mice, suggesting a disrupted bone microarchitecture and homeostasis in cancellous bone (Figure 1H, red arrows).

### Bone-Fracture Healing Was Delayed in miR-378 TG Mice

**Figure 2:**
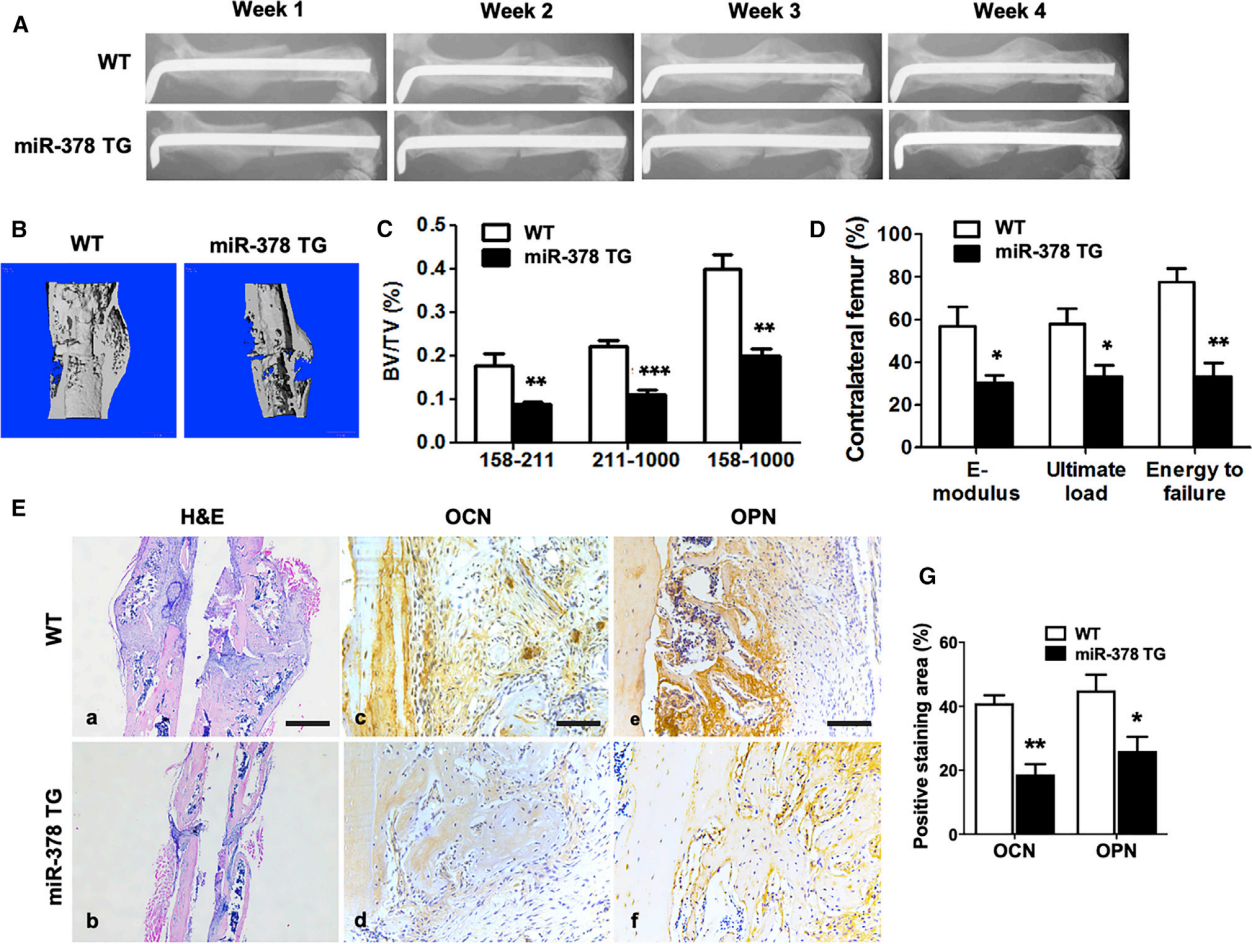
Bone Fracture Healing Was Impaired in the miR-378 TG Group (A) Representative images of X-ray radiography showed that less callus formation was found in the miR-378 TG group at week 4, and the gap in the fracture sites almost disappeared in the WT group. (B) Three-dimensional micro-CT images of the mouse femur fracture zone were taken 4 weeks after surgery, and the result confirmed that less continuous callus was found in the miR-378 TG group. (C) Micro-CT analysis was performed, and highly mineralized and newly formed calluses were reconstructed based on a different threshold. The results showed that BV/TV of total mineralized bone (threshold 158–1,000), highly mineralized bone (threshold 211–1,000), as well as new callus (threshold 158–211) in the WT group was much higher than that in the miR-378 TG groups. (D) Three-point bending mechanical testing in the miR-378 TG group showed a significant decrease of ultimate load, E-modulus, and energy to failure compared to the WT group. (E, a and b) H&E staining revealed that the miR-378 TG group has fewer calluses covered on the femur fracture site at week 4 postsurgery, and the bone remodeling was less vigorous than the WT group. Scale bar, 800 μm. (E, c–g) Immunohistochemistry staining and semiquantitative analysis revealed less OCN and OPN expression in the miR-378 TG group. Scale bars, 100 μm. (E, g) Age: 3 months; n = 10; ∗p < 0.05, ∗∗p < 0.01, ∗∗∗p < 0.001 versus WT group.

Following the observed abnormal bone tissues in miR-378 TG mice, an established mouse femoral fracture model was performed to compare the healing process of bone fracture between miR-378 TG and WT mice. The healing process was assayed by X-ray examination, and the representative images showed that less callus formation was observed in the miR-378 TG group compared with the WT group during the healing process (Figure 2A). At week 4, the gap in the fracture sites disappeared in the WT group, whereas it appeared in miR-378 TG group, indicating that the fracture-healing process was delayed in miR-378 TG mice. The 3D reconstructed images of micro-CT also confirmed the lower bone mass in the TG mice at week 4 (Figure 2B). The value of bone volume over total volume (BV/TV) was calculated, and the results exhibited a significant decrease of newly formed mineralized bone (threshold 158–211), highly mineralized bone (threshold 211–1,000), and total mineralized bone (threshold 158–1,000) in the miR-378 TG group (Figure 2C). Furthermore, three-point bending mechanical testing was performed, and results showed that the miR-378 TG group had a significant decrease in ultimate load, E-modulus, and energy to failure (Figure 2D). H&E staining of the fracture-healing position revealed that the fracture site of the WT group was covered by more calluses, and the bone remodeling was more vigorously compared with the TG group (Figure 2E, a and b). Moreover, by immunohistochemistry (IHC) staining and semiquantitative scoring, the decreased osteocalcin (OCN) and osteopontin (OPN) expression was also observed in the miR-378 group (Figure 2E, c–g), suggesting an impaired effect of miR-378 on bone formation and remodeling.

### miR-378 Suppressed Osteogenic Differentiation of MSCs

**Figure 3:**
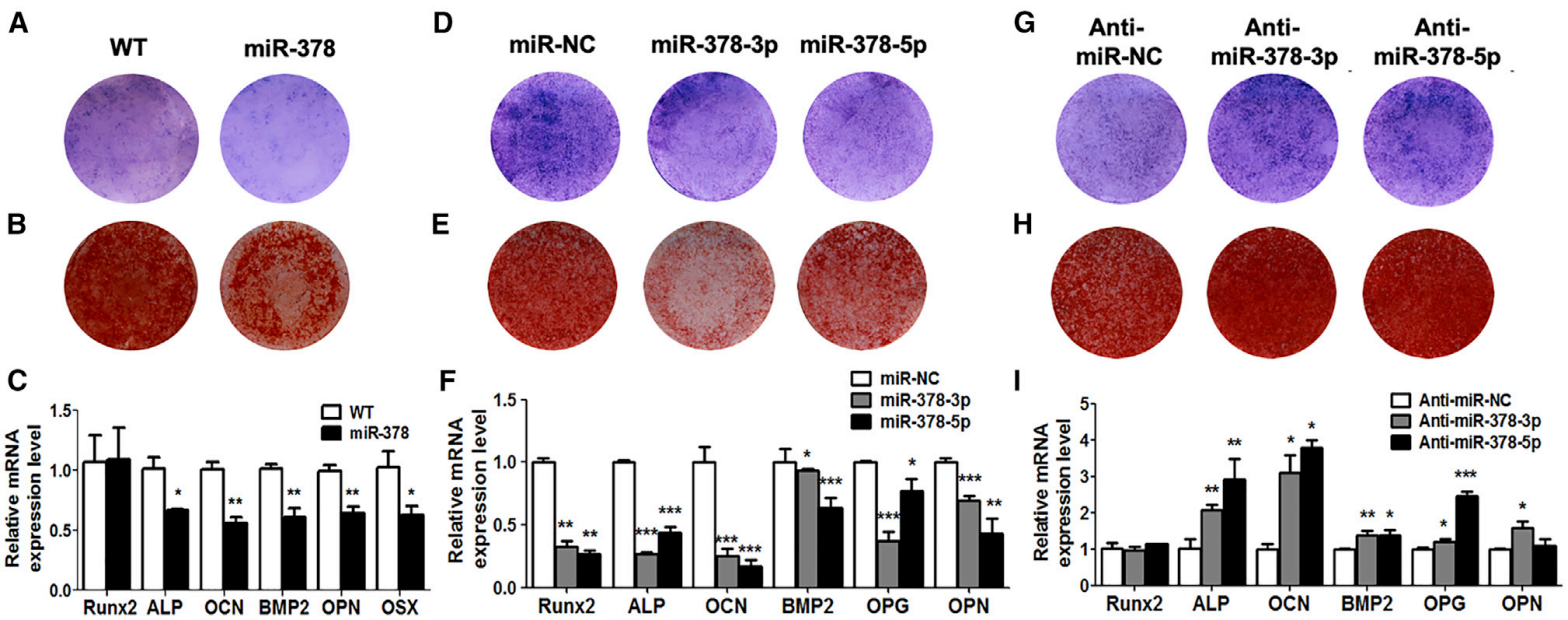
miR-378 Suppressed Osteogenesis of MSCs (A–C) Under osteogenic induction, ALP activity (A), calcified nodules (B), and osteogenic marker gene expression (C) were all suppressed in MSCs derived from miR-378 TG mice (n = 3; ∗p < 0.05, ∗∗p < 0.01, ∗∗∗p < 0.001 versus WT group). (D–F) ALP activity (D), calcified nodule formation (E), and osteogenic marker genes expression (F) were all decreased by miR-378-3p or miR-378-5p mimics (n = 3; ∗p < 0.05, ∗∗p < 0.01, ∗∗∗p < 0.001 versus miR-NC group). (G–I) ALP activity (G), calcified nodule formation (H), and osteogenesis marker gene expression (I) were promoted by anti-miR-378-3p and anti-miR-378-5p (n = 3; ∗p < 0.05, ∗∗p < 0.01, ∗∗∗p < 0.001 versus anti-miR-NC group).

To analyze the involvement of miR-378 during osteogenesis, we next investigated the osteogenic potential of MSCs derived from miR-378 TG mouse. Under the osteogenic-inductive condition, alkaline phosphatase (ALP) activity, an early marker of osteogenesis, was lower in MSCs derived from the miR-378 TG group when compared with that from the WT group at day 3 (Figure 3A), and the TG group had fewer mineralized nodules compared to that of the WT group at day 14 (Figure 3B). Furthermore, the expression of osteogenic marker genes, including ALP, OCN, OPN, osteoprotegerin (OPG), BMP2 and osterix (Osx), was significantly repressed in the TG group, whereas the Runt-related transcription factor 2 (Runx2) is not much different between the two groups at day 7 (Figure 3C).

To identify further the function of miR-378 in osteoblast differentiation, miR-378 mimics and the antagonist were also introduced into human bone marrow MSCs under the osteogenic-inductive condition. ALP activities were suppressed by miR-378-3p and miR-378-5p mimics at day 3 (Figure 3D), and fewer calcium nodules were observed in miR-378-3p and miR-378-5p groups at day 14 (Figure 3E). The expression of osteogenic marker genes, including Runx2, ALP, OCN, BMP2, OPN, and Osx was significantly suppressed by miR-378-3p and miR-378-5p mimics (Figure 3F). On the other hand, when the antagonists of miR-378-5p and miR-378-3p were introduced, the ALP activity (Figure 3G), mineralized nodules (Figure 3H), and osteogenic marker gene expression were obviously increased except for Runx2, which was not elevated by miRNA inhibitor application (Figure 3I). All of these data demonstrate that miR-378 may directly regulate the osteogenesis of MSCs, thereby affecting bone development.

### Wnt6 and Wnt10a Were Real Targets for miR-378 in MSCs

**Figure 4:**
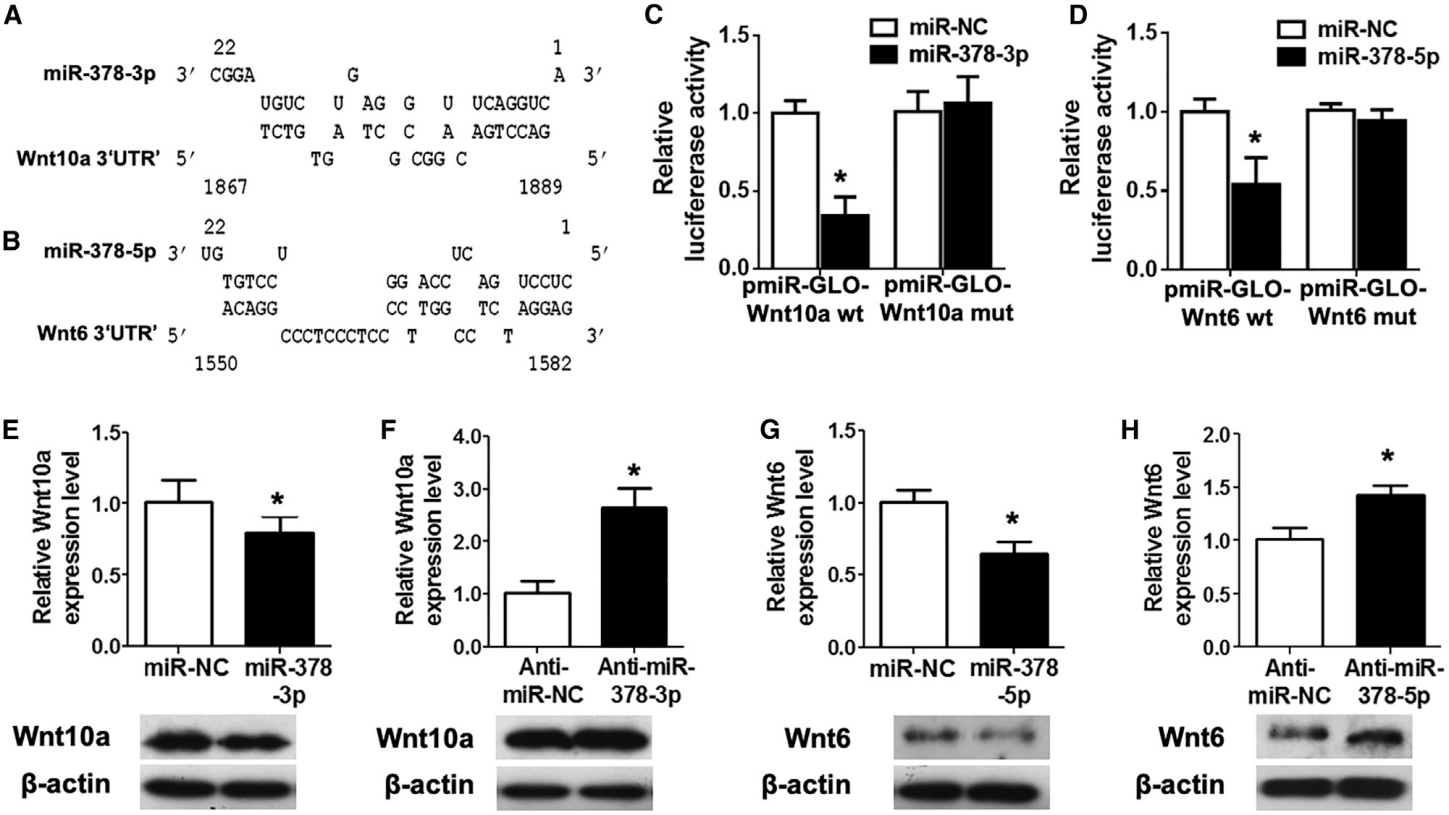
Wnt10a and Wnt6 Were Bona Fide Targets for miR-378-3p and miR-378-5p, Respectively (A and B) Schematic diagrams of the interaction between miR-378-3p (A) and miR-378-5p (B) transcripts and their direct target genes Wnt10a (A) and Wnt6 (B). (C and D) miR-378-3p (C) or miR-378-5p (D) was cotransfected with the Wnt10a (C) or Wnt6 (D) luciferase reporter into HEK293 cells, and the luciferase activity was measured (n = 3; ∗p < 0.05 versus miR-NC group). (E and F) Expression of Wnt10a was suppressed by miR-378-3p mimics (E), whereas promoted by its antagonist (F) at mRNA and protein levels (n = 3; ∗p < 0.05 versus miR-NC or anti-miR-NC group, respectively). (G and H) Expression of Wnt6 was reduced by miR-378-5p mimics (G), whereas enhanced by its antagonist (H) at mRNA and protein levels (n = 3; ∗p < 0.05 versus miR-NC or anti-miR-NC group, respectively).

miRNAs function as regulators in multiple biological activities through directly targeting protein-coding genes. In terms of the suppressive effect of miR-378 on osteogenesis, we tried to characterize the candidate target genes of this miRNA. Putative miRNA binding sites were identified by online miRNA binding-site prediction tools, including miRBase, miRanda, and TargetScan. The putative target genes of miR-378 isoforms were retrieved from three databases, and target genes intersected by all three databases were selected. Among the candidates predicted by bioinformatics analyses, the Wnt family members Wnt6 and Wnt10a were found to be the most promising candidates for their osteogenesis and bone formation potentials.^11^ Their predicted binding sites were shown in Figures 4A and 4B. To validate their direct interaction, the binding and mutated sites into the 3′ untranslated region (3′ UTR) of Wnt6 and Wnt10a were inserted into the pmiR-GLO vector to generate the luciferase reporter vectors. Cotransfection of miR-378 isoforms with two luciferase reporters was performed, and it was shown that miR-378-5p and miR-378-3p mimics dramatically suppressed the luciferase activity of these luciferase reporters of Wnt6 and Wnt10a, respectively, and mutations on their binding sites successfully abolished the suppressive effects (Figures 4C and 4D). With miR-378-3p and miR-378-5p mimics transfection, the expression of Wnt10a and Wnt6 were both significantly reduced in human MSCs at the mRNA and protein levels (Figures 4E, 4G, and S2). On the contrary, both miR-378-3p and miR-378-5p inhibitors promoted the expression of Wnt10a and Wnt6 at mRNA and protein levels, respectively (Figures 4F, 4H, and S2).

### Wnt/β-Catenin Signaling Was Involved in miR-378-Mediated Osteogenesis

**Figure 5:**
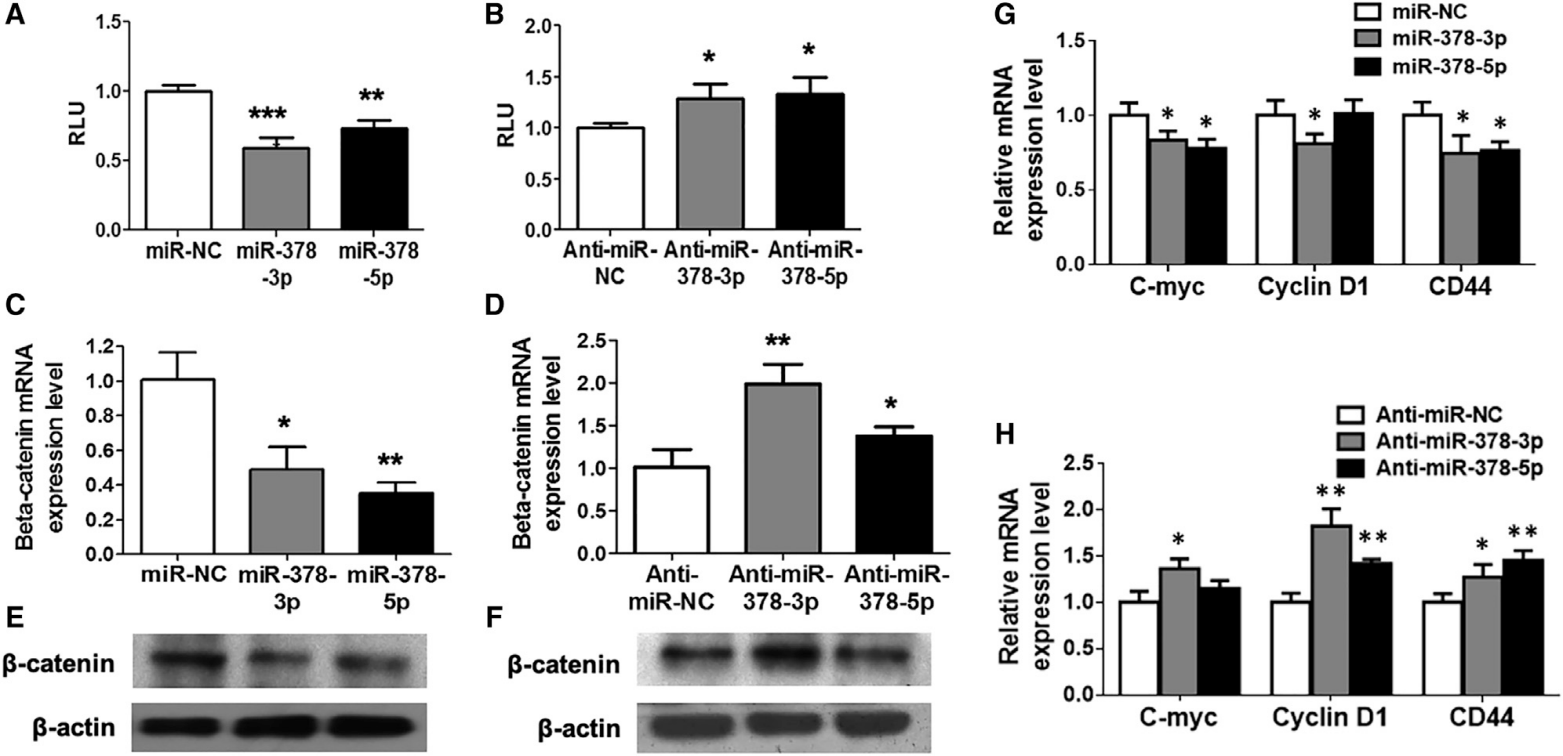
Wnt/β-Catenin Signaling Was Suppressed in miR-378-Mediated Osteogenesis (A and B) miR-378 mimics suppressed (A), whereas their antagonist promoted (B) the TOPflash luciferase activity (n = 3; ∗p < 0.05, ∗∗p < 0.01, ∗∗∗p < 0.001 versus miR-NC or anti-miR-NC group, respectively). (C–F) Expression of β-catenin was evaluated with miR-378 mimics or the antagonists’ treatment at mRNA (C and D) and protein (E and F) levels (n = 3; ∗p < 0.05, ∗∗p < 0.01 versus miR-NC or anti-miR-NC group, respectively). (G and H) miR-378 mimics (G) suppressed, whereas their antagonists (H) activated several downstream target genes of β-catenin (n = 3; ∗p < 0.05, ∗∗p < 0.01 versus miR-NC or anti-miR-NC group, respectively).

With the consideration that both Wnt6 and Wnt10a were members of Wnt family, we next investigated whether Wnt/β-catenin signaling could be involved in the miR-378-mediated osteogenesis. The Wnt signaling reporter TOPflash, which contains three β-catenin binding sites for the T cell factor/lymphoid enhancer factor (TCF/LEF), was introduced. The results showed that both miR-378-3p and miR-378-5p mimics were repressed, whereas their inhibitors activated luciferase activities (Figures 5A and 5B). Consistent with these results, we also found that miR-378-3p and miR-378-5p mimics were suppressed, whereas their inhibitors promoted β-catenin expression at mRNA and protein levels (Figures 5C–5F and S2). Furthermore, several downstream transcriptional targets, such as c-Myc and cluster of differentiation (CD)44, were significantly downregulated by ectopic expression of miR-378 isoforms. Cyclin D1 was repressed by miR-378-3p but not miR-378-5p (Figure 5G). Cyclin D1 and CD44 were significantly upregulated by miR-378 inhibitors, whereas c-Myc expression was promoted by the miR-378-3p inhibitor but not the miR-378-5p inhibitor (Figure 5H).

**Figure 6:**
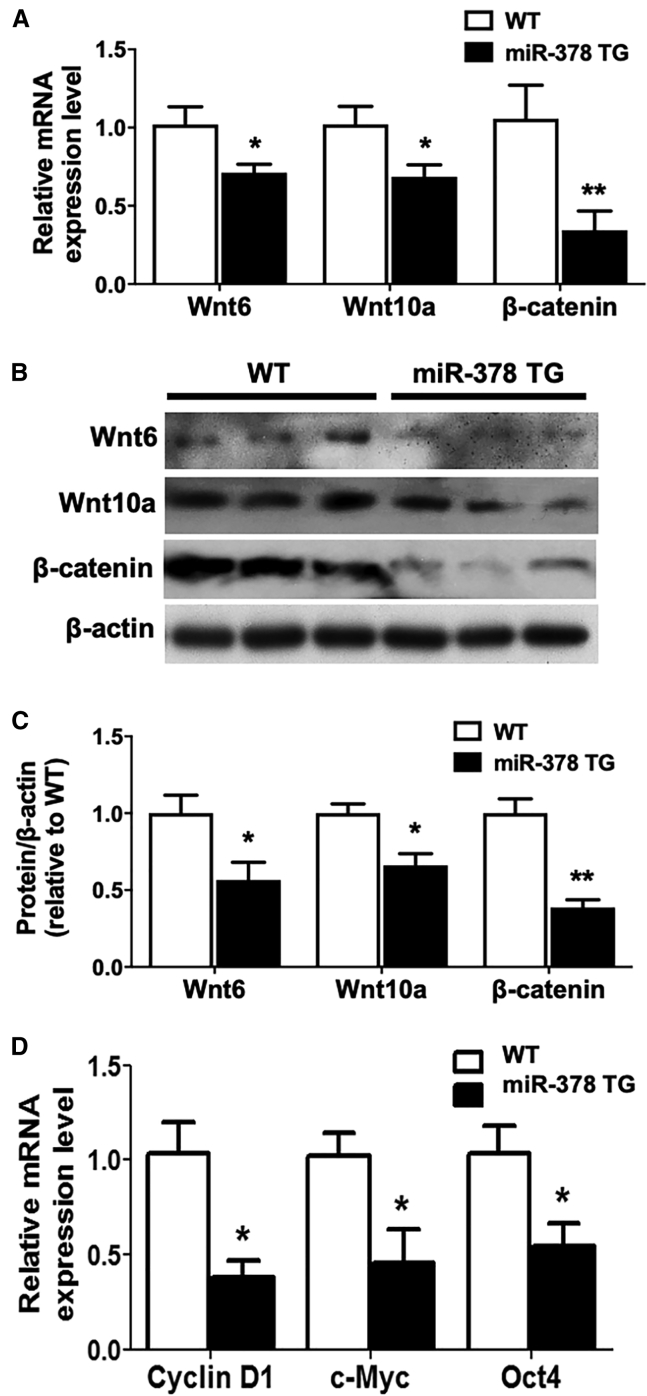
Wnt/β-Catenin Signaling Was Suppressed in miR-378 TG Mice (A) Expression of Wnt10a, Wnt6, and β-catenin was decreased in the MSCs derived from miR-378 TG mice at the mRNA level (n = 3; ∗p < 0.05 versus WT group). (B and C) Western blot (B) and semiquantitative analysis (C) revealed that Wnt10a, Wnt6, and β-catenin protein expression was suppressed in the MSCs isolated from miR-378 TG mice. (D) Several downstream target genes of β-catenin were downregulated in the MSCs derived from miR-378 TG mice (n = 3; ∗p < 0.05 versus WT group).

To confirm further the involvement of Wnt/β-catenin signaling *in vivo*, the MSCs derived from miR-378 TG mice was used for investigation. Downregulated Wnt6, Wnt10a, and β-catenin (Figure 6A) were observed in the MSCs derived from the miR-378 TG group. Consistent with the mRNA results, their protein expression was all decreased in MSCs from the TG group (Figures 6B and 6C), and the Wnt/β-catenin signaling downstream target genes, such as c-Myc, CD44, and cyclin D1, were significantly downregulated in MSCs derived from the miR-378 TG mouse (Figure 6D). All of these data suggest that Wnt/β-catenin signaling could be involved in miR-378-mediated osteogenesis. Expression levels of several other Wnt family members activating the Wnt/β-catenin signaling pathway were also downregulated in MSCs isolated from miR-378 TG mice, which revealed a broader influence of miR-378 overexpression on Wnt/β-catenin signaling-mediated osteogenesis (Figure S3).

### Short Hairpin (sh)-miR-378 Modified MSCs’ Accelerated Bone-Fracture Healing *In Vivo*

**Figure 7:**
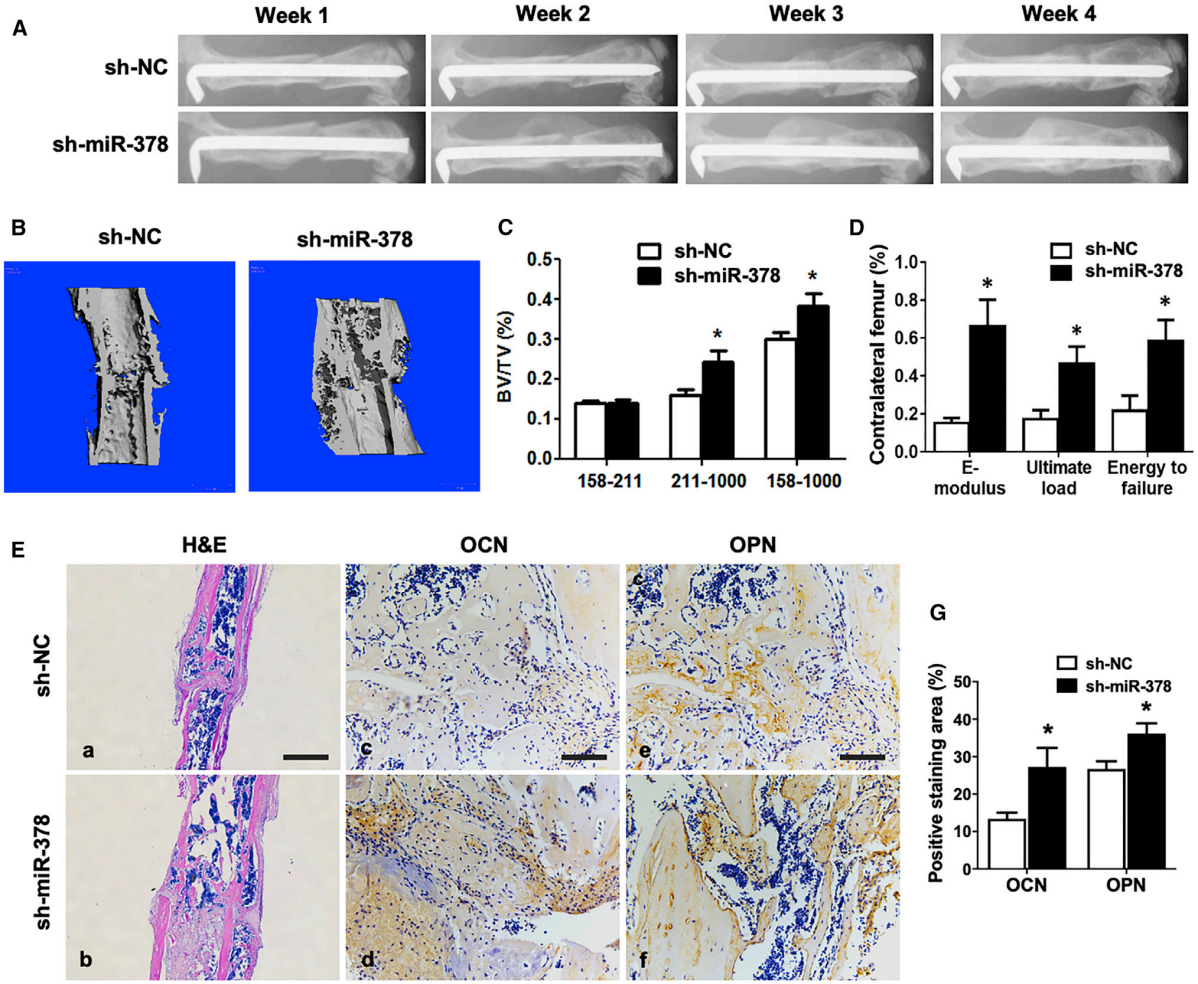
sh-miR-378-Mediated MSCs Accelerated Bone-Fracture Healing in Mice (A) X-ray radiography was taken during the course of fracture healing. Representative images showed that the fractured site was covered by more calluses, and bone remodeling was more vigorous in the sh-miR-378 group. (B) Three-dimensional micro-CT images showed a better fracture-healing effect condition and more calluses in the sh-miR-378 group. (C) Statistical diagram of BV/TV calculated from micro-CT results was displayed (age: 3 months; n = 10; ∗p < 0.05 versus sh-NC group). (D) Three-point bending mechanical testing at week 4 postsurgery was performed, and the statistical data were displayed (n = 10; ∗p < 0.05 versus sh-NC group). (E, a and b) H&E staining revealed more callus covered in the femur fracture site of the sh-miR-378 group at week 4. Scale bar, 800 μm. (E, c–g) Immunohistochemistry staining and semiquantitative analysis revealed more OCN and OPN expressed in the new bone zone in the sh-miR-378 treatment group by immunohistochemistry staining. Scale bars, 100 μm.

To investigate further the putative therapeutic effect of the miR-378 inhibitor on promoting bone formation, the femoral fracture model was constructed using miR-378 TG mice. On the other hand, the lentivirus particles were produced and infected with mouse MSCs. The miR-378 shRNA delivering lentivirus (sh-miR-378)- or negative control shRNA lentivirus (sh-NC)-infected MSCs were locally injected into the fracture sites at day 3 postsurgery. The X-ray examination results showed that the gap in the fracture sites nearly disappeared at week 4 in the sh-miR-378 group compared with the control group (Figure 7A). Further micro-CT analyses showed more newly mineralized calluses in the sh-miR-378 group (Figure 7B). Besides, the value of BV/TV indicated more highly mineralized and total mineralized bone in the sh-miR-378 group, whereas the newly formed bone showed no much differences between two groups (Figure 7C). Mechanical testing was performed at week 4, and the results showed that the E-modulus, ultimate load, and energy to failure were all significantly increased in the sh-miR-378 group (Figure 7D). The sh-miR-378- and sh-NC-infected MSCs could express the green fluorescent protein (GFP) (Figure S4A), and the GFP fluorescence of the slides confirmed the localization of these infected MSCs at the fracture callus (Figure S4B). The H&E and IHC staining was performed to evaluate the histological properties of newly mineralized bone tissues. As shown in Figure 7E, the fracture site of the sh-miR-378 group was covered by more calluses, and the bone remodeling was more vigorously compared with control group at week 4 postsurgery (Figure 7E, a and b). Moreover, the increased OCN and OPN expression was also observed in the sh-miR-378 group (Figure 7E, c–g), which suggests a rescue effect of sh-miR-378 on bone formation and remodeling of mice with miR-378 deficiency.

## Discussion

With the aging of global population, osteoporosis has been becoming a serious public health problem, which is characterized by the decreased bone density and subdued strength, eventually leading to an increased risk of fracture. In the present study, miR-378 was found to suppress osteogenic differentiation *in vitro* and impair bone formation and the fracture-healing process as well *in vivo*, indicating that miR-378 may be a potential therapeutic target for bone diseases.

As a conserved miRNA, miR-378 is located in the first intron of peroxisome proliferator-activated receptor γ coactivator-1β (PGC-1β),^12^ and it is widely expressed in many tissues, including adipose, skeletal muscles, myocardium, etc. miR-378 was originally identified as an oncogene promoting vascular endothelial growth factor (VEGF) expression in human nasopharyngeal carcinoma;^13^ it was then proven to participate in a variety of biological processes, such as cancer metastasis and differentiation.^14^^,^^15^ miR-378 was strongly upregulated during adipogenic differentiation and positively regulated adipogenesis.^16^, ^17^, ^18^ As for osteogenesis, One study reported that miR-378 promoted osteogenic differentiation,^8^ whereas another study demonstrated that miR-378 suppressed it.^10^ Therefore, the exact function of miR-378 on osteogenesis remains unclear. Recently, Zhu’s group^19^ reported that skeletal muscle mass was significantly reduced in TG mice globally overexpressing miR-378 (TG) compared with that in the WT mice. In the present study, the abnormal bone tissues and impaired bone quality were observed in the miR-378 TG mouse, and moreover, the bone-fracture healing was delayed in the femoral fracture model of this TG mouse. Our study first indicated the *in vivo* activity of miR-378 in regulation of bone development by using this TG animal model.

The *in vitro* effect of miR-378 on MSC osteogenesis was further examined in this study. The 3-[4,5-dimethylthiazol-2-yl]-2,5 diphenyl tetrazolium bromide (MTT) assay was first performed to compare the proliferation activity of bone marrow MSCs from WT and miR-378 TG mice; the result concluded that miR-378 could not alter MSC proliferation (Figure S5A). Under the osteogenic-inductive conditions, MSCs, derived from miR-378 TG mice, showed weak potential of osteogenic differentiation compared with that from WT mice. Furthermore, we found that miR-378 mimics suppressed, whereas their inhibitors could promote, osteogenic differentiation of human MSCs. All of these provide strong support for the impaired bone formation in miR-378 TG mice. Consistent with our study, miR-378 inhibited osteogenesis of the mouse osteoblast cell line MC3T3-E1 cells.^10^ miR-378, secreted by osteoclast, was also discovered to be increased in exosomes of patients with bone metastases compared to healthy controls, and the expression level was correlated with bone metastasis burden.^20^ Based on the previous reports and our results, miR-378 may be a negative regulator of osteogenesis and bone regeneration.

As for the molecular mechanism of miR-378, it has been reported that miR-378 mediated metabolic homeostasis in skeletal muscle via the Akt1/FoxO1/PEPCK pathway. IGF1R signaling pathway was also reported to be involved in miR-378-mediated muscle regeneration. Wnt/β-catenin signaling was critical for normal bone and tooth formation and development.^21^ This pathway is essential for multiple biological activities, including osteogenesis. Various studies have demonstrated that miR-378 could regulate Wnt/β-catenin signaling; i.e., miR-378 could increase neural stem-cell differentiation through Wnt/β-catenin signaling;^22^ A colon cancer study also revealed that miR-378 attenuates malignant phenotypes of colon cancer cells via suppressing the Wnt/β-catenin pathway.^23^ More importantly, miR-378a-3p could suppress Wnt/β-catenin signaling in hepatic stellate cells via targeting Wnt10a.^24^ In the current study, two Wnt family members, Wnt6 and Wnt10a, were identified as the targets of miR-378, and overexpressed miR-378 could suppress their expression, thus resulting in inactivating Wnt/β-catenin signaling. As members of the Wnt gene family, Wnt10a could induce MSC osteoblastogenesis by activating and stabilizing the downstream β-catenin expression and inducing Wnt/β-catenin signaling,^11^ and Wnt6 could act synergistically with BMP9 to induce Wnt/β-catenin signaling, as well as MSC osteogenic differentiation.^25^ Moreover, Wnt6 promoted Runx2 promoter activity directly and stimulated osteogenesis.^26^ In *in vivo* studies, Wnt6 and Wnt10a were also revealed to be pivotal characters in bone development. For example, Wnt6 is expressed during long bone development,^27^ whereas the expression level of Wnt10a was also revealed downregulated in Runx2 knockout mice,^28^ as well as bone marrow MSCs isolated from ovariectomy-induced osteoporosis mice.^29^ These data further supported that Wnt6 and Wnt10 are in the bone metabolism, and downregulated Wnt6 and Wnt10a were highly related to bone-disorder disease. Taken together, previous research and our research results all indicated that miR-378 directly suppressed Wnt6 and Wnt10a mRNA expression and hence, represses Wnt/β-catenin signaling, as well as osteogenic differentiation of MSCs.

To investigate further the *in vivo* therapeutic effect of miR-378, sh-miR-378-modified MSCs were applied to an established mouse femoral fracture model for bone-fracture treatment. Our results demonstrated that local administration of the miR-378 inhibitor-modified cells promoted bone formation and improved mechanical properties of the fractured femur. The micro-CT examination showed a significant increase of newly formed calluses and total mineralized BV in the sh-miR-378 group. Furthermore, more vigorous bone formation and bone remodeling were observed in the sh-miR-378 group by histological analyses. Therefore, these results suggest an accelerated effect of sh-miR-378 on fracture healing *in vivo*.

In summary, our data demonstrated that miR-378 could impair the bone formation *in vivo* and suppress the osteogenesis *in vitro*. Two Wnt family members, Wnt6 and Wnt10a, were identified as novel targets of this miRNA. miR-378 led to the repression of the two targets, which eventually inactivated the Wnt/β-catenin pathway and hence, suppressed osteogenesis. Therefore, mR-378 may be a potential novel therapeutic target, and the knowledge gained from this study will provide insight for developing a new cell therapy strategy to bone diseases.

## Materials and Methods

### Plasmid Generation and Cell Transfection

This study was approved by the Ethics Committee of Chinese University of Hong Kong and performed in accordance with the Code of Ethics of the World Medical Association. The miR-378 TG mice were developed and kindly provided by D.-h.Z. (Chinese Academy of Medical Sciences).^19^ The genotyping characterization of miR-378 TG mice was performed following the protocol from Zhu. et al.’s^19^ research group, and the result was described in Figure S6. miR-378 mimics and inhibitors were purchased from GenePharma (Shanghai, China). Human Wnt10a and the Wnt6 3′ UTR sequence were subcloned into the pmiR-GLO vector. All of the cDNA sequences were obtained by database searching (NCBI: https://www.ncbi.nlm.nih.gov/). sh-miR-378 lentivirus plasmid was designed and constructed by GenePharma (Shanghai, China). miRNA mimics and inhibitors, as well as DNA plasmids, were transfected using transfection reagent Lipofectamine 3000 (Invitrogen, USA) following the manufacturer’s instruction.

### Cell Culture and Osteo Induction

The human embryonic kidney 293 (HEK293) cells were purchased from ATCC and cultured in Dulbecco’s modified Eagle’s medium (DMEM), supplemented with 10% heat-inactivated fetal bovine serum (FBS) and 1% penicillin/streptomycin/neomycin (PSN). The human bone marrow-derived MSCs were isolated from bone marrow, which aspirated from healthy donors. Mouse MSCs were isolated from bone marrow of 3-month-old miR-378 TG mice, the WT mice at the same age served as a NC. Both human and mouse bone marrow was flushed out into a 10-cm cell-culture dish using minimum essential medium alpha (MEMα) plus 10% heat-inactivated FBS and 1% PSN. The culture was kept in a humidified 5% CO_2_ incubator at 37°C for 72 h when nonadherent cells were removed by changing the medium. MSCs were characterized using flow cytometry for phenotypic markers, including MSC-positive markers CD44-fluorescein isothiocyanate (FITC) and CD90-phycoerythrin (PE) and negative markers CD31-FITC and CD45-FITC following the previous protocol^6^ (Figures S5B–S5E). A trilineage differentiation assay was also performed, according to the previous protocol, and the histochemical staining result demonstrated the osteogenesis, adipogenesis, and chondrogenesis activity of isolated MSCs (Figures S5F–S5H).

To initiate osteogenic differentiation, the MSCs were seeded in a 12-well plate and grown up to 80% confluence, and 10 nM dexamethasone (Sigma-Aldrich, USA), 50 μg/mL ascorbic acid (Sigma-Aldrich, USA), and 10 mM glycerol 2-phosphate (Sigma-Aldrich, USA) were added into the culture medium. The differentiation medium was replaced every 3 days.

### ALP Activity and Alizarin Red Staining

Mouse or human MSCs were seeded in 24-well plates at a density of 2 × 10^5^ cells per well, and osteogenic differentiation was induced when cells reach 80% confluency. For the alizarin red S staining, the mouse or human MSCs were washed with PBS and fixed with 70% ethanol for 30 min. The MSCs were then stained with 2% alizarin red S staining solution for 10 min. The stained calcified nodules were scanned using Epson Perfection V850 (Seiko Epson, Japan). The ALP activity was measured and analyzed following the published protocol.^6^

### Establishment of Stable Cell Lines

sh-miR-378-mediated mouse MSCs were generated using a lentivirus-mediated gene-delivery system, as previously described.^6^ The supernatant medium containing lentivirus particles was purchased from GenePharma (Shanghai, China). The mouse MSCs were infected with the lentiviral particles by the addition of hexadimethrine bromide (Sigma-Aldrich, USA). Lentivirus-infected mouse MSCs were selected by G418 (Sigma-Aldrich, USA) at 500 μg/mL. After antibiotics selection for around 7 days, remained cultured cells were collected, and knockdown of miR-378 was confirmed by qRT-PCR examination.

### RNA Extraction and qRT-PCR Examination

Total RNA of the cultured cells was harvested with RNAiso Plus reagent (TaKaRa, Japan) following the manufacturer’s instruction. After RNA extraction, cDNAs were reversely transcribed from RNA samples by PrimeScript RT Master Mix (TaKaRa, Japan). The Power SYBR Green PCR Master Mix (Thermo Fisher Scientific, USA) was applied for the quantitative real-time PCR of target mRNA detection using ABI 7300 Fast Real-Time PCR Systems (Applied Biosystem, USA). The relative fold changes of candidate genes were analyzed by using the 2^−ΔΔCt^ method.

### Western Blot Analysis

Total protein of harvested cell was lysed using radioimmunoprecipitation assay (RIPA) buffer (25 mM Tris-Cl, pH 8.0, 150 mM NaCl, 0.1% SDS, 0.5% sodium deoxycholate, 1% NP-40), supplemented with cOmplete Mini Protease Inhibitor Cocktail (Roche, USA), and the soluble protein was collected by centrifuge at 14,000 rpm for 10 min at 4°C. Soluble protein fractures were then mixed with 5× sample loading buffer (Roche, USA) and boiled for 5 min. To perform western blotting analysis, the protein samples were subjected to SDS-PAGE gel and electrophoresed at 120 V for 2 h. After that, the protein from SDS-PAGE gel was electroblotted onto a polyvinylidene fluoride (PVDF) membrane at 100 V for 1 h at 4°C. The membranes were then blocked with 5% nonfat milk and probed with the following antibodies: β-catenin (1:3,000; BD Biosciences, USA), Wnt6 (1: 1,000; Sigma, USA), Wnt10a (1:1,000), and β-actin (1:4,000; Sigma, USA). The results were visualized on the X-ray film by Kodak film developer (Fujifilm, Japan). Integrated gray values of each band were measured using ImageJ (NIH, USA).

### Luciferase Assay

Dual-luciferase assay was performed according to the instructions of dual-luciferase assay reagent (Promega, USA) with some modifications. Briefly, HEK293 cells were seeded in a 24-well plate, and the cells were allowed to grow until 80% confluence. Cells were then transfected with TOPflash or a constructed pmiR-GLO luciferase reporter together with miR-378 mimics or antagonists. The pRSV-β-galactoside (ONPG) vector was cotransfected as normalization control. The plate was placed into a PerkinElmer Victor X2 2030 multilateral reader (Waltham, MA, USA) to measure the firefly luciferase activity, as well as the β-galactosidase activity. The ratio of firefly luciferase to β-galactosidase activity in each sample was revealed as a measurement of the normalized luciferase activity. All experiments were performed in triplicate.

### Animal Surgery and Cell Local Injection

A standard mouse open transverse femoral fracture model was applied in this study.^30^ For bone-fracture-healing capacity comparison of WT and miR-378 TG mice, 10 male mice of each type at the age of 3 months were used. For the therapeutic effect of sh-miR-378 lentivirus-infected MSCs on bone-fracture healing, 20 of the miR-378 TG mice at the age of 3 months were applied. Generally speaking, the mice were carried under general anesthesia and sterilizing procedures. A lateral incision was made through shaved skin from the right lateral knee to the greater trochanter, the osteotomy was made by a hand saw at the middle site of right femur, and then a hole was drilled at the intercondylar notch by inserting a 23G needle (BD Biosciences, USA) into the bone marrow cavity to fix the fracture. The incision was closed, and the fracture was confirmed by X-ray. For the sh-miR-378 treatment study, 20 of the miR-378 TG mice were equally and randomly assigned into 2 groups after surgery: the sh-NC group and the sh-miR-378 group. The mouse MSCs infected with sh-miR-378 or sh-NC lentivirus were harvested by 0.25% trypsin and resuspended in PBS. A total of 5 × 10^5^ cells resuspended in 20 μL PBS were locally injected into the fracture site of the bone under anesthesia using a Hamilton syringe (Hamilton, USA) and a 30G 1/2 needle (BD Biosciences, USA). X-rays were taken weekly using a digital X-ray machine (Faxitron X-Ray, USA) to evaluate the fracture-healing condition. Each mouse was exposed for 6,000 ms at a voltage of 32 kV. At 4 weeks postsurgery, the mice were sacrificed, and the femurs were collected for analysis.

### Micro-CT

The structure differences of mouse femur and tibia, as well as the structural change on the fracture sites, were quantitatively assessed using micro-CT as previously described.^31^ Mice femur and tibia samples were excised, and the muscles and soft tissues were carefully removed. All of the specimens were imaged using a high-solution μCT40 (Scanco Medical, Switzerland) with a voltage of 70 kV and a current of 114 μA and 10.5 μm isotropic resolution. The gray-scale images were segmented with a low-pass filter (sigma = 1.2, support = 2) to suppress noise and a fixed threshold to perform 3D reconstruction of the mineralized bone phase. Reconstruction of low- and high-density mineralized bone was performed using different thresholds (low attenuation = 158, high attenuation = 211) following the previously published protocol with a small modification.^32^ The high-density tissues represented the highly mineralized bone, whereas the low ones represented the newly formed callus. BV, TV, and BV/TV of each specimen were recorded for analysis.

### Three-Point Bending Mechanical Testing

Mechanical testing was performed within 24 h after termination at room temperature. The femurs were loaded on a three-point bending device (H25KS; Hounsfield Test Equipment, UK) in the anterior-posterior direction with the span of the two blades set as 8 mm. The force loading point was set at the fracture site. The femurs were tested to failure with a constant displacement rate of 6 mm/min. Test of contralateral intact femur was also included as an internal control. After test, femur-loading displacement curve was generated automatically by built-in software (QMAT Professional Material testing software; Hounsfield Test Equipment). The modulus of elasticity (E-modulus), which represents tissue stiffness, ultimate load, and energy to failure, indicating tissue toughness, was obtained by previously mentioned software. The biomechanical properties of newly formed bone were further analyzed and expressed as percentages of the contralateral intact bone properties.

### Histology and IHC

All femurs and tibia were initially fixed with 10% formalin for 48 h, followed by decalcification in 10% EDTA solution for 2 weeks. The femurs and tibia were then embedded in paraffin and sliced into 5 μm sections by a rotary microtome (HM 355S; Thermo Fisher Scientific, USA). For the bone-morphology study, the femurs and tibia were sliced along their long axis in the coronal plane and short axis in the transverse plane, respectively. For the bone-fracture study, the femurs were sliced along the long axis in the coronal plane. After deparaffinization, IHC staining was performed, and the sections were stained with H&E for histomorphometric analysis. IHC staining was performed using a standard protocol as previously reported.^30^ Secretions were treated with primary antibodies to OCN (1:100; Abcam, USA) and OPN (1:100; Abcam, USA) overnight at 4°C. The horseradish peroxidase-streptavidin system (Dako, USA) was applied for IHC signal detection, followed by counterstaining with hematoxylin. The images of positive-stained cells in the fracture site were captured using a light microscope (Leica, Cambridge, UK). The analysis of the positive-stained cell area was performed using NIH ImageJ software.

### Statistical Analysis

Experimental data are expressed as mean ± standard deviation. Data statistical analysis was performed using Student’s t test and one-way analysis of variance (ANOVA). The results were considered to be statistically significant when p <0.05.

## Author Contributions

G.L. and J.-f.Z. spearheaded and supervised all the experiments. G.L., J.-f.Z., D.-h.Z., and L.F. designed research. L.F., L.S., Z.-m.Y., T.-y.W., H.-x.W., W.-p.L., and Y.-f.L. conducted experiments. L.F. and J.-f.Z. analyzed data. D.-h.Z. provided materials. L.F., G.L., J.H.T.L., and J.-f.Z. prepared the manuscript. All authors reviewed and approved the manuscript.

## Conflicts of Interest

The authors declare no competing interests.

## Supplemental Information

Document S1. Figures S1–S6

Document S2. Article plus Supplemental Information
