# Supplementary material for: MicroRNA-378 Suppressed Osteogenesis of MSCs and Impaired Bone Formation via Inactivating Wnt/β-Catenin Signaling
Source: Mol Ther Nucleic Acids. 2020 Jul 15;21:1017–28. doi: 10.1016/j.omtn.2020.07.018 (PMC7452050; doi:10.1016/j.omtn.2020.07.018)
Supplement: Document S1. Figures S1–S6 [file mmc1.pdf]

OMTN, Volume *21*

Supplemental Information

MicroRNA-378 Suppressed Osteogenesis of MSCs and Impaired Bone Formation via Inactivating Wnt/β-Catenin Signaling

Lu Feng, Jin-fang Zhang, Liu Shi, Zheng-meng Yang, Tian-yi Wu, Hai-xing Wang, Wei-ping Lin, Ying-fei Lu, Jessica Hiu Tung Lo, Da-hai Zhu, and Gang Li

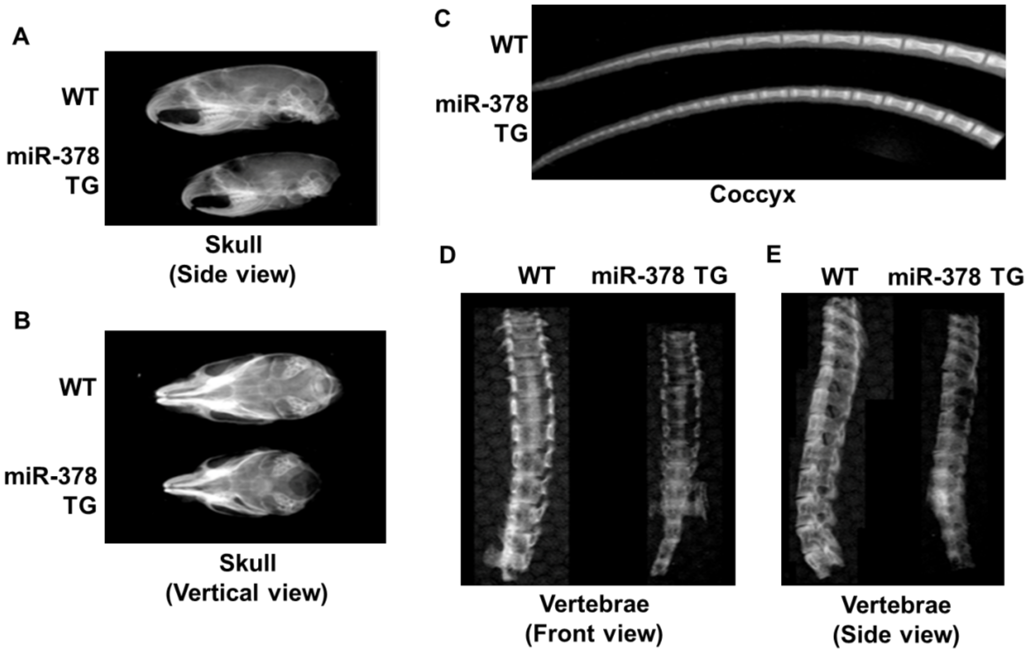

Supplementary Figure 1. The bone phenotype of skull (**A** for side view and **B** for vertical view), tail **(C)** and spine (**D** for front view and **E** for side view) of miR-378 TG mice and their wild-type (WT) mice were examined by digital radiography.

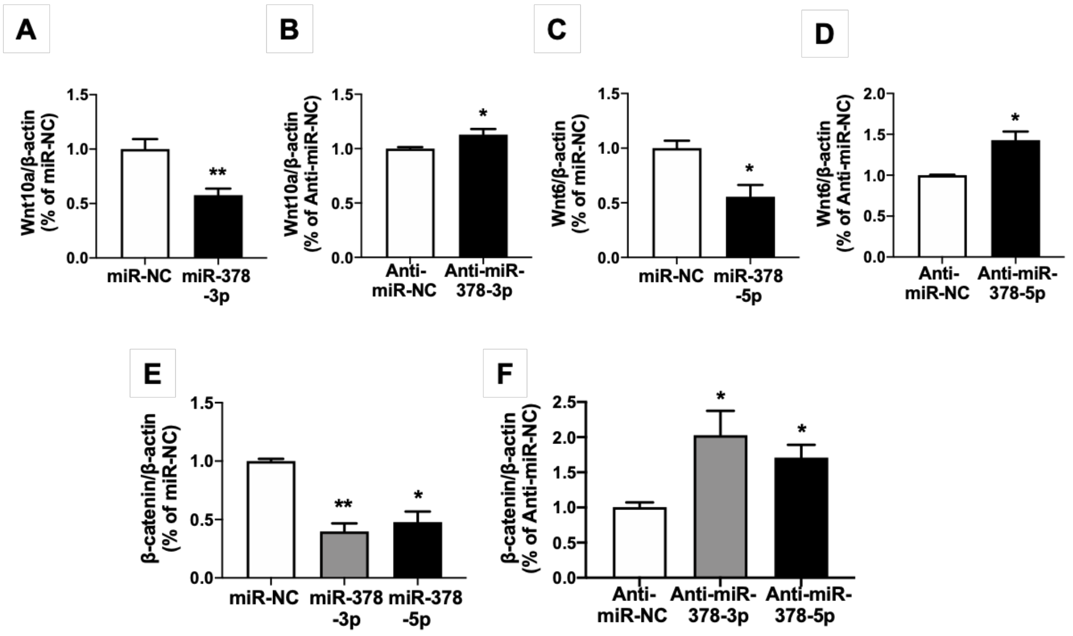

Supplementary Figure 2. Semiquantitative densitometry analyses of representative Western blot signals revealed in Figure 4E **(A)**, Figure 4F **(B)**, Figure 4G **(C)**, Figure 4H **(D)**, Figure 5E **(E)**, and Figure 5F **(F)** (n = 3; *P < 0.05, versus miR-NC or anti-miR-NC group, respectively).

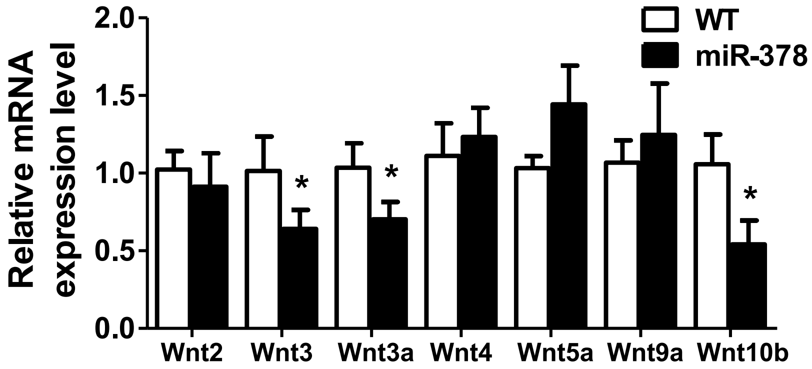

Supplementary Figure 3. The mRNA expression level of Wnt family members which could activate Wnt/β-catenin signaling pathway revealed by Real-time PCR. (n = 3; *P < 0.05, versus WT group).

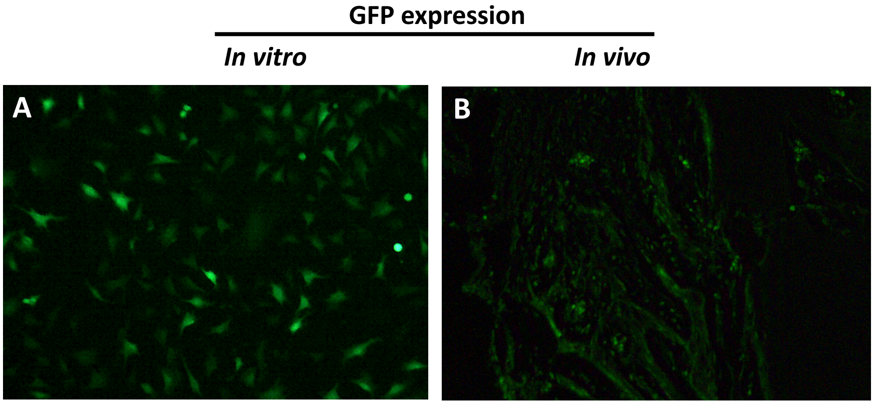

Supplementary Figure 4. The GFP expression was detected in sh-miR-378 infected BMSCs

A, scramble or sh-miR-378 infected BMSCs *in vitro* (GFP positive cells) **B,** the sh-miR-378 infected MSCs in fracture callus 4 weeks after bone fracture (GFP positive cells).

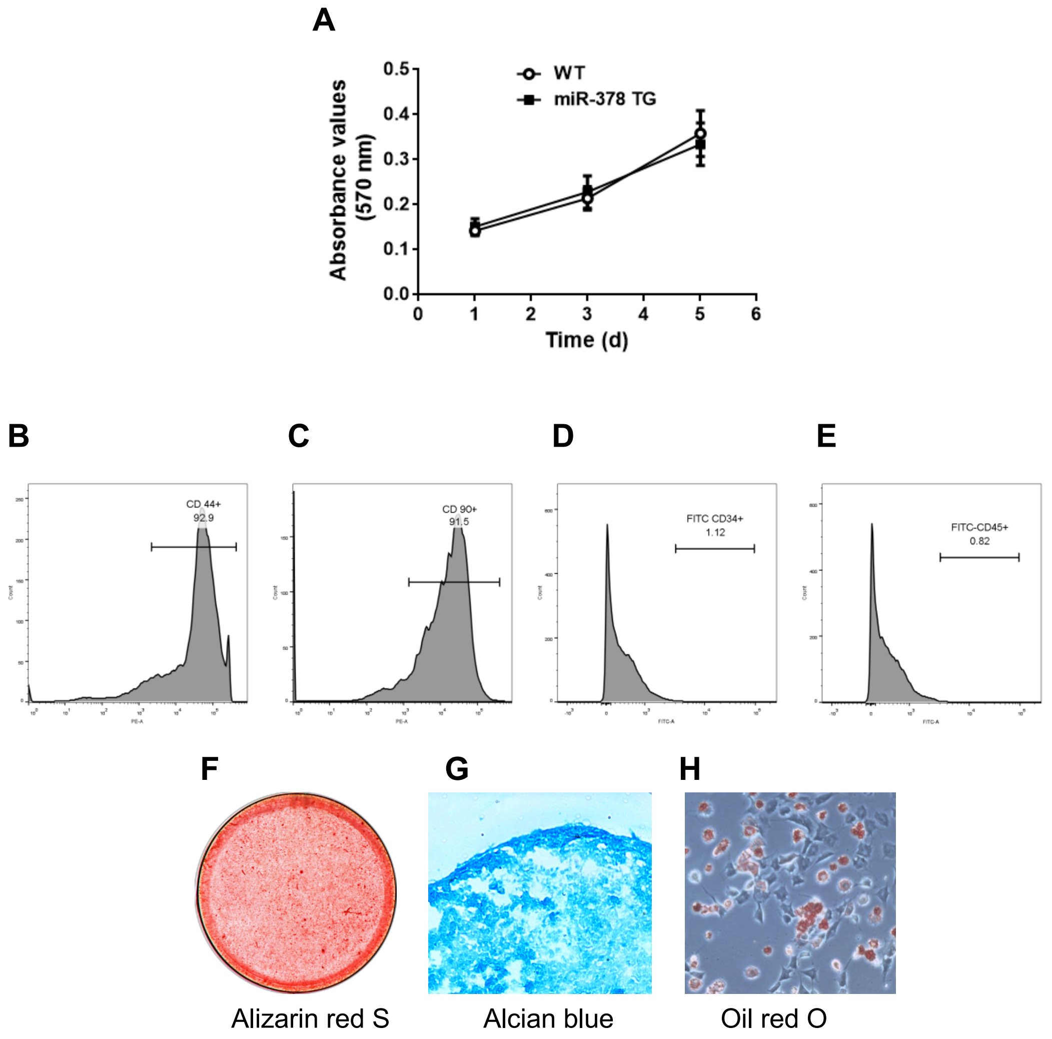

Supplementary Figure 5. Confirmation of mouse bone marrow MSCs by using flow cytometry and study of MSC proliferation activities

A, MTT assay of MSCs isolated from WT and miR-378 TG mice. The two cells showed no obviously different proliferation activity. **B&C,** flow cytometry analysis results showed that these cells were positive for MSC markers CD44 (B) and CD90 (C). **D&E,** cells were negative for endothelial cell marker CD34 (D) and haematopoietic cell marker CD45 (E). **F-H,** MSCs displayed osteogenic (F), chondrogenic (G) and adipogenic activities (H).

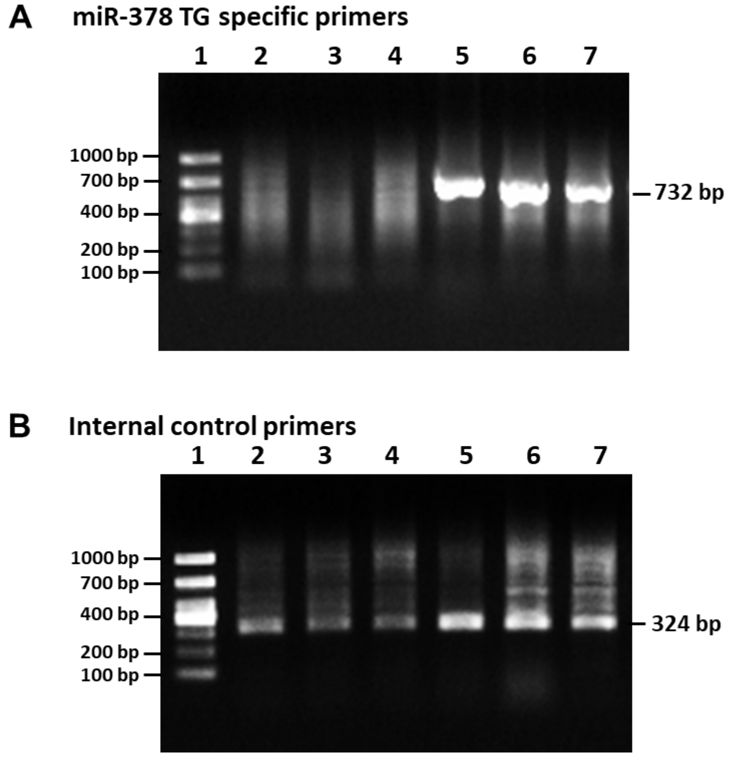

Supplementary Figure 6. Gel electrophoresis of PCR products using specific primers for the genotyping of miR-378 TG mice. **A,** miR-378 TG specific primers: 732 bp. **B,** internal control primers: 324 bp. Lane 1: 100 bp DNA ladder. Lane 2-4: WT mice genotype. Lane 5-7: miR-378 TG mice genotype.
